# Practical Notes on Diagnosis and Treatment

**Published:** 1910-05-21

**Authors:** 


### Practical Notes on Diagnosis and Treatment.

### Nocturnal Incontinence.

A dose of antipyrin (phenazone) at bedtime has had good effect in these cases, and may be continued for months without harm.—*Dr. L. Phillips.*

### Senile Tuberculosis.

Spontaneous pain (apart from movement) is suggestive of cancer rather than tubercle, while redness over diseased bone is indicative of tubercle rather than cancer. The occurrence of senile tuberculosis is more frequent than is generally supposed.

### Asthma in Children.

Nocturnal paroxysms may be prevented by phenacetin or bromides at bedtime, with small doses of heroin during the day.—*Dr. Cautley.*

### Chicken Pox in the Adult.

In chicken pox in early life there is, as a rule, no severe febrile onset, but in cases of adults the disease occasionally comes on with high temperatures, severe headaches, and even pain in the back. —*Dr. Foord Caiger.*

